# Treatment of Concurrent Thrombotic Thrombocytopenic Purpura and Graves' Disease: A Report on Two Cases

**DOI:** 10.1155/2018/5747969

**Published:** 2018-08-09

**Authors:** Karl Lhotta, Emanuel Zitt, Hannelore Sprenger-Mähr, Lorin Loacker, Alexander Becherer

**Affiliations:** ^1^Department of Internal Medicine 3, Academic Teaching Hospital Feldkirch, Feldkirch, Austria; ^2^Central Institute for Medical and Chemical Laboratory Diagnostics, Medical University Innsbruck, Innsbruck, Austria; ^3^Department of Nuclear Medicine, Academic Teaching Hospital Feldkirch, Feldkirch, Austria

## Abstract

Graves' disease (GD) and thrombotic thrombocytopenic purpura (TTP) are autoimmune diseases caused by autoantibodies against the TSH receptor (TRAb) and the enzyme ADAMTS13. We here report on two patients with concurrent GD and TTP, who achieved sustained remission of both conditions with the TTP treatment regimen and thiamazole. Both patients suffered from relapsing TTP and were diagnosed with GD concomitantly at the time of relapse. They were treated with steroids, plasma exchange, rituximab, and thiamazole. This therapy induced complete remission of TTP. TRAb levels also decreased rapidly and both patients developed subclinical hypothyroidism three and five weeks later. Our observations suggest that TTP and GD may be concomitant and that GD possibly triggers a relapse of TTP. The combination of thyrostatic treatment and immunosuppression with PE, rituximab, and steroids is able to induce rapid and prolonged remission of GD.

## 1. Introduction

Graves' disease (GD) is the most common cause of hyperthyroidism with a lifetime risk of 3% for women and 0.5% for men [1]. The disease is caused by activating autoantibodies directed against the alpha subunit of the TSH receptor (TRAb) on thyroid follicular cells [2]. Graves' disease is currently treated with either thyrostatic drugs such as thiamazole or propylthiouracil, which block thyroid hormone synthesis, radioactive iodine, or surgical thyroidectomy [3]. Remission after medical thyrostatic therapy is achieved in about 50% of patients. Although a theoretical option, targeting autoimmunity with immunosuppression is currently not pursued in patients with the disorder. We here report on two patients with concurrent thrombotic thrombocytopenic purpura (TTP), an autoimmune disease caused by autoantibodies against the von Willebrand factor-cleaving protease ADAMTS13, and GD. Treatment consisting of steroids, plasma exchange, and rituximab induced rapid and sustained remission of both conditions.

## 2. Patient 1

A 40-year-old woman sought medical treatment because of petechia, hematuria, and headache. Laboratory analysis revealed severe hemolytic anemia with schistocytosis and thrombopenia. ADAMTS13 activity was absent (<24 ng/mL, reference range 530-800 ng/mL), but no inhibitor could be detected. A diagnosis of thrombotic thrombocytopenic purpura was made despite a negative test for anti-ADAMTS13 antibodies [4]. She made a quick recovery with steroids and daily plasma exchange (PE) using fresh frozen plasma as a substitution fluid. After one week she experienced a severe relapse with microangiopathic involvement of the brain, heart, lung, kidneys, liver, spleen, stomach, and gut. PE was performed twice daily. Altogether, the patient had 41 exchanges over a six-week period. In addition, she received two 1g infusions of rituximab. Thyroid function was normal. The patient made a complete recovery and ADAMTS13 activity remained in the normal range.

Six years later the patient experienced a relapse of her TTP, again with absent ADAMTS13 activity but undetectable inhibitor. She had mild involvement of the brain (headache), kidneys (microhematuria and albuminuria), and gut (abdominal pain). She received oral steroids (starting dose methylprednisolone: 1 mg/kg bodyweight), eleven PEs and two 1g rituximab infusions two weeks apart, and completely recovered. The patient also reported weight loss, nervousness, and increased sweating before clinical relapse. TSH was suppressed, and FT3 and FT4 were mildly elevated (Table 1). Ultrasound of the thyroid showed increased perfusion. TSH receptor antibodies (TRAb) were also elevated. A diagnosis of GD was made and thiamazole 20 mg and propranolol 20 mg twice a day were started. TRAb levels decreased by 50% after the first PE and further 50% after ten days (Table 1). Thyroid function also normalized rapidly and the patient developed peripheral hypothyroidism three weeks later. Thiamazole and propranolol were discontinued. The patient subsequently had normal thyroid function and a negative test for TRAb. Two and a half years later TRAbs were of borderline value and TSH, FT3, and FT4 remained normal. ADAMTS13 activity was in the normal range. Thyroid function is being closely monitored in order to avoid hyperthyroidism and relapse of TTP.

## 3. Patient 2

A 25-year-old female was admitted because of petechiae, hematuria, and menorrhagia. Blood tests showed hemolytic anemia and thrombopenia. ADAMTS13 activity was reduced (59 ng/mL) and an inhibitor was detectable (0.75 BU/mL, reference: <0.2 BU/mL). The patient made a quick and complete recovery with steroids, three PEs and a single 1g dose of rituximab. At that time her TSH was normal (0.98 mIU/L).

Two years later, after an uneventful pregnancy and a cesarean section, she relapsed with ADAMTS13 of 38 ng/mL and a positive inhibitor test (2.6 BU/mL). She was treated with steroids, ten PEs, and 1g rituximab followed by 0.5g after the first PE and 1g after the plasma exchange series. At that time, her thyroid function was not assessed, but TSH had been normal (0.93 mIU/L) three months earlier.

Another two years later the patient had a second relapse with severe headache and petechiae. Again, ADAMTS13 activity was reduced (128 ng/mL) and anti-ADAMTS13 antibodies were present (1.89 BU/mL). Besides, she had tachycardia of 120 beats per minute and thyroid function tests confirmed a thyrotoxicosis (Table 1). A retrospective analysis of a stored blood sample taken before the first plasma exchange showed elevated TRAbs. Sonography revealed thyroidal hyperperfusion, and pertechnetate uptake was increased upon scintigraphy. TTP was treated with methylprednisolone 1mg/kg body weight as starting dose, thirteen PEs, and 1g rituximab followed by two doses of 0.5 g. Two weeks later the TRAb titer had fallen by three-quarters. GD was treated with thiamazole 20 mg and propranolol 20 mg, each twice a day. Five weeks later the patient had developed subclinical hypothyroidism with low FT4 (Table 1). Thiamazole was reduced to 5 mg daily. Three months later the patient had reached a stable euthyroid state and thiamazole was further reduced to 5 mg on alternate days. Eight and twelve months after diagnosis of GD, TRAb levels were in the normal range below 1.75 U/L.

## 4. Discussion

We here report on two patients with concomitant TTP and GD. This combination has been described before in four case reports [5–8]. An association between both TTP [9] and GD [10] and other autoimmune diseases is well described, but concurrence of TTP and GD is not mentioned in those publications, probably because of the rarity of TTP. TTP has a low incidence of 3 cases per million persons per year [11], whereas GD is rather common with 20 to 50 cases per 100.000 [1]. In our patients TTP presented before GD and GD was associated with a relapse of TTP. Whether this is a coincidence or whether GD is indeed the cause of the TTP relapse remains unknown, but we assume a causal relationship is very likely. The nature of this causal link remains to be determined, but could be by augmented stimulation of synthesis of (possibly preformed) ADAMTS13 autoantibodies or some endothelial activation, thus triggering a TTP relapse. A causal link is also supported by one of the case reports describing remission of TTP without PE after successful treatment of GD with radioactive iodine [5].

The four previous case reports all describe good response of TTP to PE and of GD to PE and either antithyroid drugs [6, 8], radioiodine [7], or radioiodine and surgery [5]. The current therapy for relapsing TTP is a combination of steroids, PE with FFP as substitution fluid and rituximab [12, 13]. In our two patients, this therapy caused complete remission of TTP, but also rapid recovery from hyperthyroidism. One reason for that may be that PE is able to remove TRAbs, as shown in our patients, in whom TRAb levels fell rapidly (Table 1) compared to the expected normal half-life of 21 days of IgG TRAbs [14]. Number and frequency of PE sessions (11 in Patient 1 and 13 in Patient 2) were determined by TTP response to treatment. PE is also considered to be a therapeutic option in thyroid storm, not only because it removes TRAbs, but also because it lowers the pool of T3 and T4 bound to plasma proteins [15]. Rituximab is an anti-CD20 monoclonal antibody that eliminates B lymphocytes. It is used in B-cell lymphomas and autoimmune diseases such as rheumatoid arthritis or anti-neutrophil cytoplasmic antibody-associated vasculitis. In TTP, rituximab and steroids are applied to block ADAMTS13 autoantibody synthesis. It can be expected that this therapy also affects TRAb formation. Indeed, our patients quickly became TRAb-negative and remained in remission thereafter. In Patient 1, a borderline TRAb level was detected two and a half years after the attack, when recovery from rituximab-induced B-cell depletion is likely.

In addition to removal and suppression of TRAb synthesis, our patients also received standard therapy with thiamazole to block thyroid hormone synthesis. This combination therapy actually led to subclinical hypothyroidism with low FT4 levels after three and five weeks, respectively, and to normalization of TSH levels after four and six weeks, when thiamazole was able to be discontinued or the dose dramatically reduced.

Rituximab has been used in the treatment of GD and especially Graves' orbitopathy (GO) with mixed results. One small study described sustained remission in four out of ten GD patients after rituximab compared to none of the ten patients not on rituximab. Rituximab seemed to be effective in patients with low TRAb levels but had no effect on TRAb titers in addition to thiamazole [16]. Another study in relapsing patients found a persistent remission in nine out of 13 patients [17]. In addition, rituximab led to a decrease in TRAb levels. Two studies in GO compared rituximab and iv methylprednisolone. In the first study including 32 patients, rituximab was superior to iv methylprednisolone [18], whereas in the second study comprising 21 patients, it was not [19]. In both studies TRAb titers fell with rituximab. The decrease in TRAb levels induced by rituximab occurs slowly over several months [17, 20], which is comparable to the effect of antithyroid drugs on TRAb levels [21]. Rituximab, however, has been shown to specifically reduce the production of thyroid-stimulating autoantibodies, whereas methimazole has no such effect [22]. In conclusion, we report on two patients, in whom GD possibly caused a relapse of preexisting TTP. GD should be considered as a risk factor for TTP recurrence and we suggest routine assessment of thyroid function in such patients. Initiation of TTP treatment with steroids, PE, and rituximab with the addition of thiamazole caused rapid and prolonged remission of GD. This treatment regimen could also be considered, for example, in GD patients with thyroid storm. Further studies are required to prove its effectiveness and to determine the optimal dose of steroids, PE, and rituximab.

## Figures and Tables

**Table 1:** Laboratory results of Patients 1 and 2 at time of TTP diagnosis and over the course after TTP therapy.

**Patient 1**		**Patient 2**	
**TSH** (0.34.4.94 mIU/L)			

basal	<0.1	basal	<0.1
3 weeks	0.53	5 weeks	4.36
24 months	2.78	6 months	1.66
31 months	2.48	15 months	0.99

**FT3** (1.71-3.71 pg/ml)			

basal	7.00	basal	12.7
3 weeks	1.01	5 weeks	3.40
24 months	3.16	6 months	3,17
31 months	3.24	15 months	3.64

**FT4** (9.3-17.0 pg/ml)			

basal	19.7	basal	30.7
3 weeks	5.4	5 weeks	6.9
24 months	11.5	6 months	12.4
31 months	11.8	15 months	15.7

**TRAb** (<1.75 U/L)			

basal	28.3	basal	10.1
after 1. PE	5.4		
10 days	1.8	2 weeks	2.7
24 months	0.3	8 months	1.5
31 months	1.8	15 months	<0.8
